# Nonlinear Control in the Nematode *C. elegans*

**DOI:** 10.3389/fncom.2020.616639

**Published:** 2021-01-22

**Authors:** Megan Morrison, Charles Fieseler, J. Nathan Kutz

**Affiliations:** ^1^Department of Applied Mathematics, University of Washington, Seattle, WA, United States; ^2^Department of Neurobiology, University of Vienna, Vienna, Austria

**Keywords:** feed-forward control, nonlinear control, dimensionality reduction, neural network models, *C. elegans*

## Abstract

Recent whole-brain calcium imaging recordings of the nematode *C. elegans* have demonstrated that the neural activity associated with behavior is dominated by dynamics on a low-dimensional manifold that can be clustered according to behavioral states. Previous models of *C. elegans* dynamics have either been linear models, which cannot support the existence of multiple fixed points in the system, or Markov-switching models, which do not describe how control signals in *C. elegans* neural dynamics can produce switches between stable states. It remains unclear how a network of neurons can produce fast and slow timescale dynamics that control transitions between stable states in a single model. We propose a global, *nonlinear* control model which is minimally parameterized and captures the state transitions described by Markov-switching models with a single dynamical system. The model is fit by reproducing the timeseries of the dominant PCA mode in the calcium imaging data. Long and short time-scale changes in transition statistics can be characterized via changes in a single parameter in the control model. Some of these macro-scale transitions have experimental correlates to single neuro-modulators that seem to act as biological controls, allowing this model to generate testable hypotheses about the effect of these neuro-modulators on the global dynamics. The theory provides an elegant characterization of control in the neuron population dynamics in *C. elegans*. Moreover, the mathematical structure of the nonlinear control framework provides a paradigm that can be generalized to more complex systems with an arbitrary number of behavioral states.

## 1. Introduction

The emergence of large scale neural recordings across model organisms is revolutionizing the potential for the theoretical modeling of how neuron population dynamics is accomplished. With the recent advancements in whole brain imaging technologies for the nematode *C. elegans* (Schrödel et al., 2013; Prevedel et al., 2014; Nguyen et al., 2016), the relationship between neural activity and behavioral outcomes can be studied in a holistic fashion. More precisely, *C. elegans* provides a unique opportunity to quantify neuron population dynamics as it has only 302 neurons whose stereotyped electro-physical connectivity map (connectome) is known from serial section electron microscopy (White et al., 1986; Chen et al., 2006). We show that the neuron population dynamics of the *C. elegans* nematode can be characterized by a global nonlinear control model which matches experimental measurements. Moreover, it provides a general mathematical framework that illustrates how non-linearity can be exploited to produce a global model of neuron population dynamics and how it can be readily applied to more complex model organisms.

Data from *C. elegans* neural recordings show that high-dimensional neuronal activity produces dominant, low-dimensional patterns of activity across the connectome, with interpretable clusters (Kato et al., 2015; Kutz et al., 2016; Roberts et al., 2016; Kunert-Graf et al., 2017; Liu et al., 2017; Fieseler et al., 2018). Previous analysis of behavioral and calcium imaging data can be categorized within three different modeling paradigms, each with their own strengths and weaknesses: Markov models, switching linear dynamical systems, and models with control. An overview is given in Table 1.

**Table 1:** Different modeling paradigms for *C. elegans* with experimental implications.

**Paradigm**	**Parameters**	**What it models**	**Implications**	**References**
Markov	<10	Clusters and transitions	Differentiate macro-scale behaviors	Gallagher et al., 2013
Linear (switching)	>1,000	Local linear dynamics in neuron-activity spaces	Connection between behavioral clusters and neural dynamics within them	Linderman and Adams, 2014; Linderman et al., 2016, 2019; Costa et al., 2019
Linear (controlled)	>100	Global linear dynamics and control inputs	Disentangle intrinsic dynamics and transition mechanisms	Fieseler et al., 2020
Nonlinear (controlled)	<10	Global nonlinear dynamics and control inputs	Can model different classes of transitions	This work

The most well-established methodology is the Hidden Markov Model (HMM) which has been used for decades (Roberts et al., 2016). This paradigm simplifies data into clusters, and assumes instantaneous transitions between them. Such models have been used to uncover different macro-level behaviors in animals that are characterized by different stabilities of individual behaviors, such as the difference between roaming and foraging (Arous et al., 2009). While these models capture the differential stability of various behaviors, they are statistical models and do not show how the dynamics in the network generate these transitions.

Recent work extends the HMM paradigm to trajectories in neuron space (Linderman and Adams, 2014; Linderman et al., 2016, 2019; Costa et al., 2019). This paradigm models the neural network as having distinct linear dynamics within different states, allowing a connection between behavior-level HMMs and neural trajectories at the cost of many more parameters. Mathematically, this is given by $\overset{∙}{\text{x}}={\text{A}}_{i}\text{x}$, where **x** is the state space, the dot represents time differentiation, and *i* refers to multiple segmented state spaces. However, these models are fundamentally local, and it is unclear whether switching between different states can be biologically achieved.

A recent paradigm has built a global model for calcium imaging dynamics by including a control signal (Fieseler et al., 2020). This is given by the equations $\overset{∙}{\text{x}}=\text{A}\text{x}+\text{B}\text{u}$ where **x** is the state space, the dot represents time differentiation and **u** is the control signal. The global matrices **A** and **B** characterize the intrinsic dynamics, and how actuation forces these dynamics respectively. This paradigm allows the control signals to be studied as independent objects, hypothesizing a separation between the intrinsic dynamics of the network and the mechanisms that cause transitions. However, this work uses a linear framework, which requires that there is only a single fixed point at the origin. From this perspective, all behaviors except one are merely long-lived and do not have their own fixed point.

In contrast to linear models which can only support a single fixed point in the dynamics, nonlinear models offer a more flexible architecture for control, especially in systems like the *C. elegans* where multiple behavioral states appear to be stable. We show that with minimal parametrization, we can construct a global nonlinear model of the underlying *C. elegans* control structure. Our nonlinear control model removes the need for multiple linear models and provides a parsimonious, global control framework parameterized by only a few parameters and consistent with experimental observations. Nonlinear control theory takes the form $\overset{∙}{\text{x}}=f\left(\text{x}\right)+g\left(\text{u}\right)$ where *f*(·) specifies the nonlinear dynamics and *g*(·) specifies the actuation on the underlying dynamics. This provides a theoretical framework for circumventing many of the standard limitations inherited from linear control theory. This comes at the expense of provable controllability criteria which can be rigorously stated in linear theory. A fundamental benefit of nonlinear control theory is that one can posit an underlying model with *multiple fixed points* where *f*(**x**_*j*_) = 0 and *j* = 1, 2, ⋯ , *N*. In the context of neuron population dynamics and *C. elegans*, these *N* fixed points correspond to distinct behavioral states, i.e., forward or backward motion. Thus, instead of regressing to the matrices **A** and **B** in constructing a linear model, we instead posit a global model whose features are consistent with experimental observations (Kato et al., 2015). We propose a model of the form

(1) $$\begin{array}{r} x^{\prime }=F\left(x,\beta \right)+u\left(t\right) \end{array}$$

where *F*(***x***, β) represents the intrinsic nonlinear dynamics containing multiple stable states; β parameterizes fluctuations in intrinsic dynamics that may occur over long timescales. Fast-timescale control signals *u*(*t*) control state location by applying feed-forward control to the intrinsic dynamics.

Nonlinear control has been used to induce and describe transitions between stable attractors in the nonlinear dynamics of other biological networks (Purnick and Weiss, 2009). In synthetic biology, researchers have created nonlinear, bistable gene regulatory networks in *Escherichia coli* that can be toggled between different states with the use of control signals (Gardner et al., 2000). Control applied to key nodes can induce a nonlinear system to converge to a desired state rather than an undesired state (Cornelius et al., 2013). Stochasticity is also a mechanism for control and is used by organisms to regulate transcription (Kepler and Elston, 2001). Previous work has considered control in bistable systems implemented via feed-forward control pulses (Sootla et al., 2015, 2016, 2018) and through analysis of saddle points in the system (Trotta et al., 2012). We hypothesize that the neural network of *C. elegans* uses nonlinear control mechanisms, such as those previously explored, to transition between various stable states and vary its transition probabilities. Feed-forward control signals could take the form of activity in dedicated control neurons, such as sensory neurons, but they may have a distributed representation. We aim to represent the local and non-local neural activity that, holistically, implements effective control in the nematode, as a single, low-dimensional time series *u*(*t*).

Our model has the flexibility to describe *C. elegans* dynamics under a wide variety of internal states and environmental stimulus. Quantitative work on postural analysis of the behaving *C. elegans* has demonstrated there is low-dimensional structure on the level of individual movements and body bends (Stephens et al., 2008, 2011). The statistics of how often these movements happen show the presence of a few discrete clusters (Pierce-Shimomura et al., 1999; Wakabayashi et al., 2004; Arous et al., 2009; Churgin et al., 2017), or a spectrum of behavioral strategies (Gallagher et al., 2013; Hums et al., 2016) that are appropriate in different environments and may even be different between individuals (Moy et al., 2015). Recent modeling work has used a conceptual or data-driven model of multiple fixed points in the neuron population phase space (Roberts et al., 2016; Chen et al., 2019). However, it remains unclear how statistics of transitions between behaviors can be controlled by global parameters, or how individual trajectories through state space are affected in these cases. Our model is able to reproduce the changes in statistics between the large-scale roaming and dwelling behaviors via changing a single global parameter. In addition, this model reproduces observed short time-scale bursts of reversals interspersed with extremely short-lived forward states.

Our model further could be used to produce testable hypotheses of the effects of neuromodulators on global dynamics. Much work has been done in recent years to extend the understanding of internal *C. elegans* dynamics beyond simple synaptic connections to include additional layers, particularly the slower dynamics of neuromodulators (Komuniecki et al., 2014; Bentley et al., 2016). Specifically, single molecules and simple neuronal circuits have been found to change global statistics related to fundamental behaviors, most clearly the frequency of reversal initiation (Wakabayashi et al., 2004; Arous et al., 2009; Flavell et al., 2013; Bhattacharya et al., 2014; Hums et al., 2016; Lim et al., 2016; Churgin et al., 2017; McCloskey et al., 2017). Because our model is able to reproduce macro-scale behavioral changes with a single parameter, we hypothesize that there may be a correspondence between some neuromodulators and our model parameters. As we will show, our global nonlinear model is minimally parameterized and provides a parsimonious representation of the neuron population dynamics of the *C. elegans* nematode. These parameters have suggestive connections to experimental work, and some may correspond to one or more neuromodulators. This mathematical framework is general, and can be readily applied to more complex model organisms.

## 2. Results

We introduce a nonlinear global model with control for the low dimensional activity of *C. elegans* neuron population dynamics that captures the behavioral dynamics that we aim to model. Any model of this data must satisfy the following requirements: (1) the general structure of the model must support the two fixed points observed in the data, (2) the model must be flexible enough to accommodate the full range of variability observed in *C. elegans*, and (3) the model must be minimally parameterized such that the modulation of only a few parameters can generate this full range of variability. We start by observing the structure of the data and posit a general model whose parameters can be tuned to generate activity that is analogous to the activity observed in the data. We then explore how experimentally observed changes in *C. elegans* behavior can be explained by the modulation of single parameters.

### 2.1. Nonlinear Global Dynamical Models for *C. elegans*

**Figure 1:**
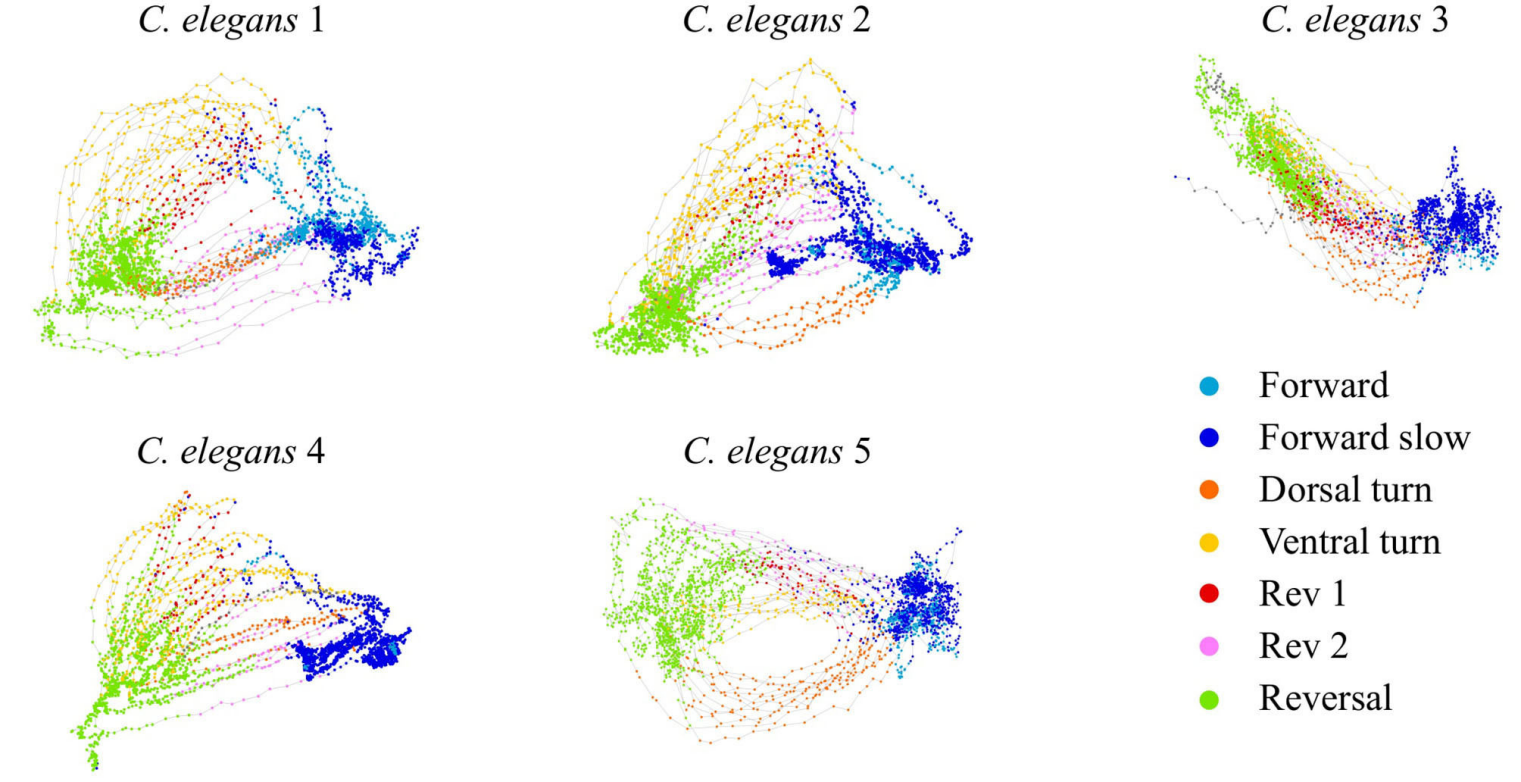
*C. elegans* neural activity in the PCA space of the first two modes. Trajectories colored by behavioral state.

We construct a generalized, low-dimensional representation of the neural activity of *C. elegans* by performing PCA on the activity of neurons in five *C. elegans* from Kato et al. (2015). We then use the first two PCA modes to represent the dynamics linked to behavior (see section 4). Distinct behaviors correspond to different regions of PCA space (Figure 1). Forward and reversal behaviors (states 1, 2, and 7) correspond to two distinct stable states in PCA space. Dorsal and ventral turn behaviors (states 3 and 4) correspond to reversal to forward transitions while rev 1 and rev 2 behaviors (states 5 and 6) correspond to forward to reversal transitions. Behavioral states during calcium imaging are determined by Kato et al. (2015) and Skora et al. (2018). We fit our general dynamical systems model to the trajectories of the dominant mode *v*_1_(*t*) which most strongly differentiates the stable states from the transition states.

This low-dimensional representation suggests a feature space for a model decomposition. Specifically, it allows us to build a control model which accurately reproduces the global dynamics with minimal parametrization. The nonlinear parsimonious and global control model takes the form

(2) $$\begin{array}{r} x^{\prime }=y\\ y^{\prime }=f\left(x,\beta \right)+\gamma y+u\left(t\right), \end{array}$$

where the nonlinear dynamics is prescribed by the cubic

(3) $$\begin{array}{r} f\left(x,\beta \right)=-\left(x+1\right)\left(x-\beta \right)\left(x-1\right), \end{array}$$

which has by construction (for *u* = 0) two stable fixed points at *x* = ±1 and a single unstable fixed point whose location is determined by the parameter β. Additionally, there is a damping parameter γ and a control input *u*(*t*). These relate to the dominant PCA modes directly, where *x*(*t*) = *v*_1_(*t*) and *y*(*t*) = *v*_2_(*t*). Due to the stochastic nature of the observed data, we additionally add stochastic terms and arrive at the system

(4) $$\begin{array}{r} dx_{t}=y_{t}dt+\sigma dW_{t}\\ dy_{t}=-\left(x_{t}+1\right)\left(x_{t}-\beta \right)\left(x_{t}-1\right)dt+\gamma y_{t}dt+u\left(t\right)dt+\sigma dW_{t}, \end{array}$$

where β and γ parameterize the cubic dynamical system, and σ and *dW*_*t*_ characterize the Brownian motion which models the noisy fluctuations observed in experiments. We chose a two dimensional model fit to the first two *C. elegans* PCA modes as this is the minimum number of modes that captures the stable state clusters as well as the variability in the transition trajectories. While a higher dimensional model would capture more of the variance in the neural activity, and a model with more parameters would increase the model fit, we prioritize minimal parameterization. Our objective is to create the lowest dimension model with the fewest number of parameters that is able to represent three features of the *C. elegans* neural activity: (1) the intrinsic stability of the neural activity underlying the forward and reversal behaviors, (2) the variability in transition trajectories, and (3) the destabilization of the stable states under the influence of feed-forward control signals, that is, control of the network's state. A nonlinear control model that is higher-dimensional, or that has more parameters, can be found using the methods outlined in Morrison and Kutz (2020).

**Figure 2:**
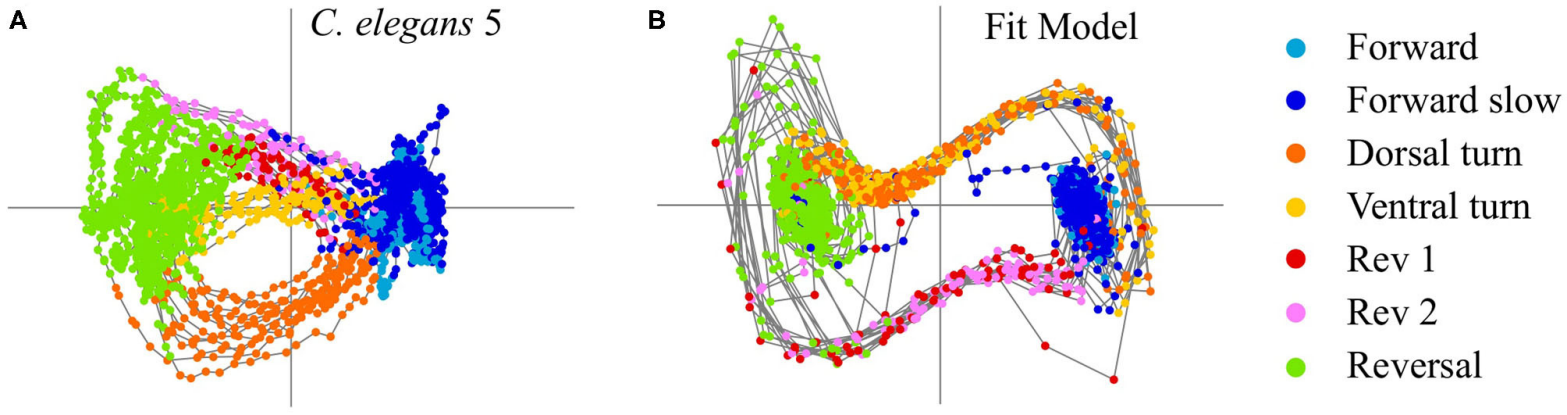
**(A)** PCA activity of *C. elegans* 5. **(B)** Dynamical systems control model fit to PCA activity of *C. elegans* 5.

**Figure 3:**
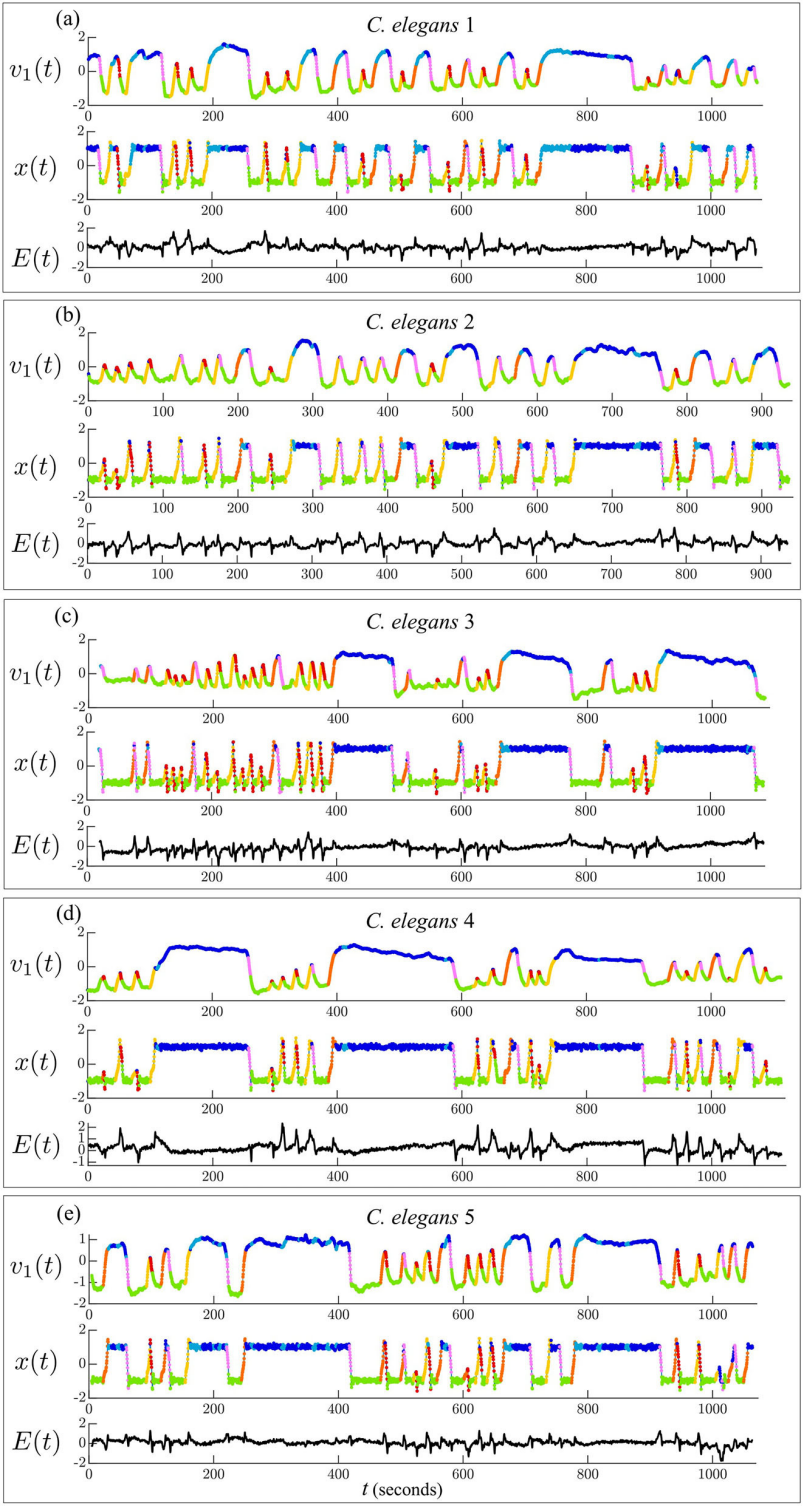
Timeseries of dominant mode of *C. elegans* neural activity [*v*_1_(*t*)] and corresponding model variable [*x*(*t*)]. Models are fit to each *C. elegans* by minimizing the error [*E*(*t*)] between the PCA and model timeseries. Trajectories are colored by behavioral state.

We fit the model parameters to the low-dimensional *C. elegans* activity by minimizing the error between the dominant PCA trajectory *v*_1_(*t*) and the trajectory of the corresponding model variable *x*(*t*) (see section 4). The labeled, behavioral states timeseries determines when the dynamical system is in the uncontrolled state *u*(*t*) = 0 for states 1, 2, and 7 (forward, forward slow, and reversal) or a transition state, *u*(*t*) = *u*_3,4_ (dorsal and ventral turns) or *u*(*t*) = *u*_5,6_ (rev 1 and rev 2 transitions). The dynamical system fitted to *C. elegans* 5 is

(5) $$\begin{array}{r} x^{\prime }=y+0.06dW_{t}\\ y^{\prime }=-\left(x_{t}+1\right)\left(x_{t}-0.11\right)\left(x_{t}-1\right)dt\\ \;-1.51y_{t}dt+0.06dW_{t}+u\left(t\right)dt, \end{array}$$

with control signal strengths *u*_3,4_ = 0.54 and *u*_5,6_ = −0.77 and timescaling parameter *dt* = 0.29. Figure 2 shows a comparison of the *C. elegans* neural activity in PCA space (Figure 2A) with the dynamical systems model reproducing this activity colored by behavioral state (Figure 2B). Model parameters for all five *C. elegans* are shown in Table 2 and a comparison of the data, model, and errors is shown in Figure 3. Across all models, β≈0.1 indicates that the model's reversal state (*x* = −1) is more stable than the forward state (*x* = 1). Because the rev 1 and rev 2 transitions are shorter than the dorsal and ventral turn transitions, stronger control signals are necessary to complete the forward to reversal transition |*u*_5,6_|>|*u*_3,4_|. A weaker forward state stability (β≈0.1) also aids in the forward to reversal transitions.

**Table 2:** Parameters for models fit to each *C. elegans* calcium imaging dataset.

***C. elegans***	**β**	**γ**	**σ**	***u*_3,4_**	***u*_5,6_**	***dt***
Id 1	0.1108	−1.5027	0.0574	0.4969	−0.7531	0.2902
Id 2	0.1124	−1.5281	0.0584	0.5059	−0.7159	0.2953
Id 3	0.1107	−1.5321	0.0584	0.5262	−0.7549	0.2951
Id 4	0.1135	−1.4830	0.0591	0.5076	−0.7292	0.3008
Id 5	0.1087	−1.5115	0.0598	0.5350	−0.7731	0.2929

*Data, model, and error timeseries shown in Figure 3*.

Figure 3 shows the timeseries of the dominant PCA mode (*v*_1_(*t*)) compared with the corresponding model variable (*x*(*t*)) for each model fit. The error over time (*E*(*t*)) shows that *E*(*t*)≈0 during the stable states 1, 2, and 7, indicating that the model executes most transitions between the forward and reversal states successfully. The error spikes during state transitions, |*E*(*t*)|>0, indicating that the model does not capture the shape of the transitions accurately due to the model's minimal parameterization.

### 2.2. Changes to a Single Parameter Reproduce Different Long-Timescale Behaviors of *C. elegans*

**Figure 4:**
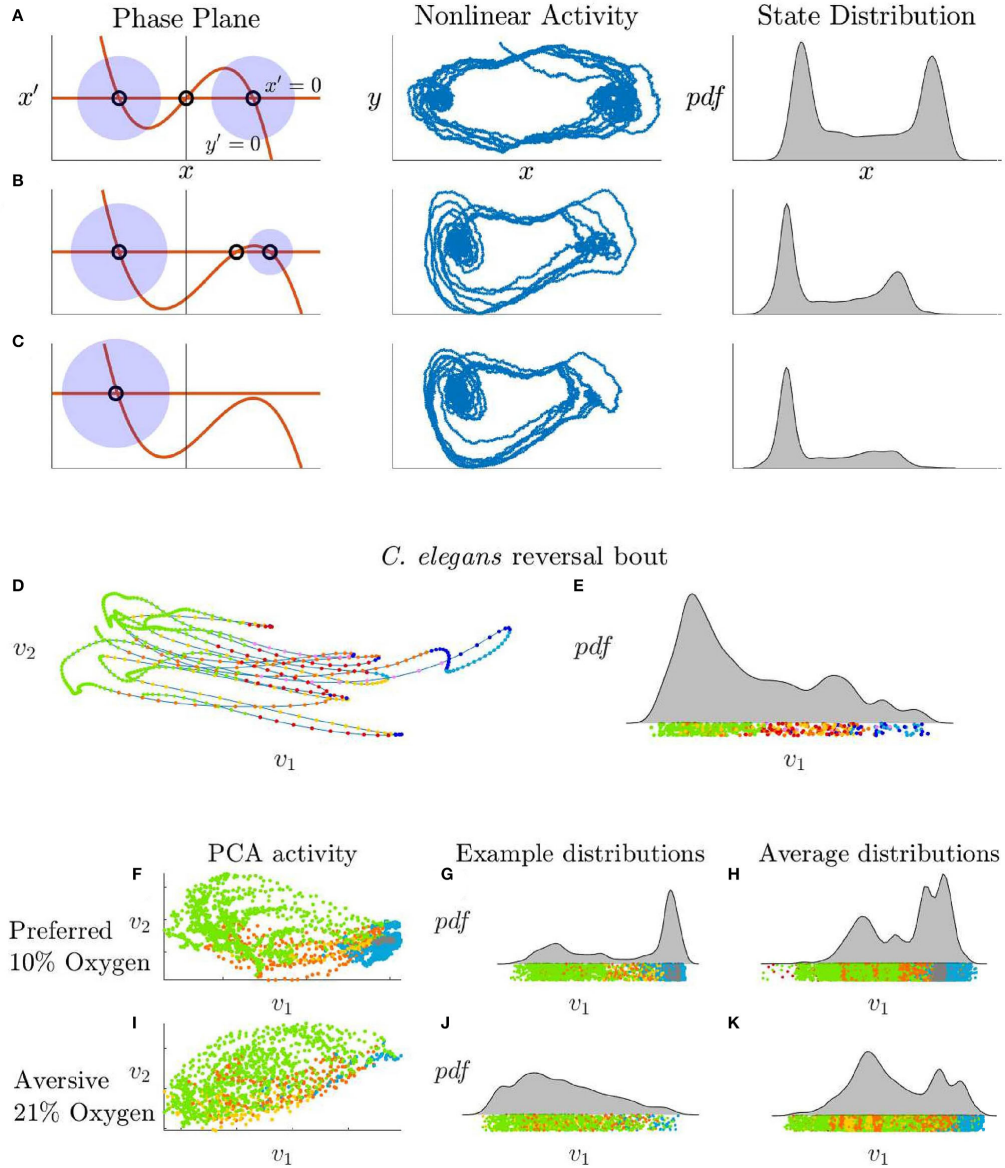
**(A–C)** Phase plane, nonlinear stochastic activity, and state distributions of Equation (4) with increasing β values. **(A)** β = 0 generates equally stable fixed points. **(B)** β = 0.6 generates a less stable fixed point which turns into a slow point as the fixed points merge. **(C)** β, *r*_2_ ∈ ℂ and the right fixed point is lost. **(D)** *C. elegans* PCA trajectory during a reversal bout and **(E)** the corresponding distribution. The forward fixed point is unstable during this interval. **(F–H)** *C. elegans* activity in a preferred 10% oxygen environment which promotes stability in the forward state compared with **(I–K)** *C. elegans* activity in an aversive 21% oxygen environment which destabilizes the forward state. **(F,G)** PCA activity and distribution of a single *C. elegans* in the preferred oxygen environment compared with the activity of this same *C. elegans* in the aversive oxygen environment **(I,J)**. Average distribution for 10 *C. elegans* in the preferred environment **(H)** compared to the aversive environment **(K)**.

As shown in the section 4, this global model has three fixed points whose stability is determined by the parameter β∈(−1, 1). The parameter γ determines the linear growth/decay rate of each fixed point. The parameter σ controls the amount of stochasticity in the system. Figures 4A–C shows the behavior of Equation (4) as a function of β for randomly generated control signals. For β = 0, there is a symmetry between the two stable fixed states corresponding to the forward and reversal state, which reproduces the long time-scale distribution of behaviors across individuals. As β approaches unity, the dynamics are skewed in favor of one of the fixed points.

The statistics of reversal length and frequency change drastically across multiple timescales during the life of a *C. elegans*. Our nonlinear control model is able to reproduce three very distinct changes in state distribution and switching frequencies seen in these experimental studies via modulation of a single parameters. The first well-studied change in these dynamics is the switch between dwelling and roaming states (Pierce-Shimomura et al., 1999; Wakabayashi et al., 2004; Arous et al., 2009; Flavell et al., 2013). Specifically, the frequency of reversals is much lower in the roaming state, which facilitates the exploration of a larger geographical area. Several neuromodulators (Flavell et al., 2013) and individual neurons (Wakabayashi et al., 2004) have been implicated in this behavioral change; some function of these chemicals or neuron activity levels might directly correspond to the model's control signal onsets and strengths *u*_3,4_ and *u*_5,6_.

### 2.3. Distinct Behaviors May Be Controlled by a Shared Mechanism

Two additional behaviors that are not known to be related can be explained using the same mechanism: spontaneous reversal bouts, and an increase in reversals in an aversive oxygen environment. We use calcium imaging data from Skora et al. (2018) to create distributions for *C. elegans* low-dimensional activity during a reversal bout and when in different controlled oxygen states (Figure 4). The reversal bout behaviors, observed in immobilized animals and shown in Figures 4D,E, are long-lived behaviors that begin in a reversal state, move into a forward motion state but then fail, and return to a reversal state several times in succession. This can be clearly related to a change in the parameter β, which controls the stability of the fixed points corresponding to forward and backward motion. A known method for experimentally destabilizing the forward state in *C. elegans* is through a modification of their environment. In an environment with a preferred oxygen level of 10%, *C. elegans* tend to have stable forward swimming behavior, Figures 4F–H. When the oxygen in their environment is increases to 21%, they exhibit more transient forward swimming behavior, Figures 4I–K, similar to the observed “reversal bouts.”

Increasing β, as shown in Figures 4B,C, reproduces this unstable forward behavior by retaining the stochastic control signals that would normally transition the system to a forward motion state, but by reducing the stability of that fixed point so that the neural trajectory immediately falls off and returns to a reversal state. We hypothesize that β may also have a biologically correlated neuromodulator or set of neuromodulators and that stabilization of this modulation system would remove the reversal bout phenomenon.

An additional testable prediction is that some subset of neurons correlated with forward motion (e.g., the AVB and RIB pairs) or the ending of reversals (e.g., the SMDD, SMDV, and RIV pairs) may be responsible for stabilizing the forward state and others may be key for initializing the state. Opto-genetic manipulation of the “initiating” neurons without the “stabilizing” neurons should simply produce a failed forward initialization, as seen in the natural reversal bout. Similarly, inhibition of the stabilizing neurons should make forward motion an inaccessible state.

## 3. Discussion

We have produced the first global, nonlinear control model that can capture the dominant features of low-dimensional neural data. Our work demonstrates how the *C. elegans* neural network could control its global dynamics via perturbations to fixed point stability and feed-forward control signals. This model provides a control theory mechanism for switches in stochastic switching models. Our model also extends previous work by explaining incomplete or unsuccessful switching seen in reversal bouts as a change in the stability of the underlying fixed point. This model is minimally parameterized and changes in several parameters can reproduce changes in behavioral distributions akin to that of known neuro-modulators, thus producing a unifying framework for analyzing various changes in distributions of behavior at multiple timescales. In addition, the framework for building this model can be extended to other complex systems with more behavioral states which are defined by fixed points as discussed in Morrison and Kutz (2020).

Several modeling strategies have been used to model *C. elegans* behavioral and neural dynamics, and they can be classified in two ways: direct models of the trajectories in neuron space (Linderman and Adams, 2014; Linderman et al., 2016, 2019; Fieseler et al., 2020), and abstract Markov models (Roberts et al., 2016). The former has the advantage of describing neuron-level dynamics at the cost of many parameters, generally hundreds. On the other hand, Markov models do not make specific predictions about neurons or trajectories on the low-dimensional manifold, but generally have a small number of very interpretable parameters. Our model combines the strengths of both approaches, producing a model of dynamics that is both directly connected to neural activity and fits only 6 parameters. It is unclear if these parameters have biological correlates, but the fact that modulating them produces known behavioral outcomes suggests areas for future experimental work.

This modeling strategy has a few limitations. In particular, the entire model was constructed and fit using the first two PCA modes, which only account for 18–23% of the variance in the data. The dominant modes capture the dominant global dynamics; however, there are many secondary structures captured by the later modes such as transient or sparse activity. The fast-timescale signals that control the global dynamics may be captured in the activity of these higher modes. It is almost certainly true that important activity is contained in higher PCA modes, particularly when trying to incorporate more complex behaviors. In addition, it is unclear that PCA modes are the correct basis for producing models whose behaviors have biological correlates. Work regarding an interpretable choice of basis is ongoing, with nonlinear embeddings offering more flexible possibilities (Lusch et al., 2018; Champion et al., 2019).

Connected to this issue, the model does not differentiate between ventral and dorsal turns or between transitions rev 1 and rev 2. Transition paths are not clearly separable in the first two PCA modes, even though they are clearly mutually exclusive at the level of muscle activation. A model with more variables would be able to differentiate between these different transition paths from the forward to reversal state and from the reversal to forward state. Extending our framework to incorporate more subtle and complex behaviors is the subject of ongoing work.

The modeling strategy proposed in this paper used polynomials to design fixed points and the transitions between them. Even if the “true” function form is more complex, polynomials can be considered a Taylor expansion approximation of those dynamics. However, no attempt was made to explicitly derive this functional form from neuron-level non-linearities, or to include information from the known connectome (White et al., 1986). A derivation from first principles would be an exciting advance and we hope that our model, as one possible macro-scale model, can facilitate this type of theoretical development.

## 4. Methods

We construct a nonlinear control model for *C. elegans* by fitting the parameters of a general dynamical systems model with control to low-dimensional *C. elegans* neural activity. We reduce the dimension of the neural trajectories with PCA and use non-convex optimization to fit the trajectory of the dominant PCA mode to the corresponding dynamical systems model variables.

### 4.1. Dimension Reduction

*C. elegans* have been proposed to have seven different behaviors—forward motion, forward slow, dorsal turn, ventral turn, reversal 1, reversal 2, and sustained reversal (Kato et al., 2015). Further references to the forward behavior denote both the forward motion and forward slow states, and references to the reversal behavior denote the sustained reversal state. We used *C. elegans* calcium imaging data collected for Kato et al. (2015) to produce the low-dimensional activity shown in Figure 1 and the model fits shown in Figures 2, 3. We used calcium imaging data collected for Skora et al. (2018) to produce the low-dimensional activity and distributions shown in Figure 4. We used data from five different *C. elegans* from Kato et al. (2015), as their neural patterns expressed activity corresponding to different labeled behaviors. We achieved a low-dimensional representation of the activity by performing *principal component analysis* (PCA) on the time series data and focusing on the activity of the first two PCA modes.

**Figure 5:**
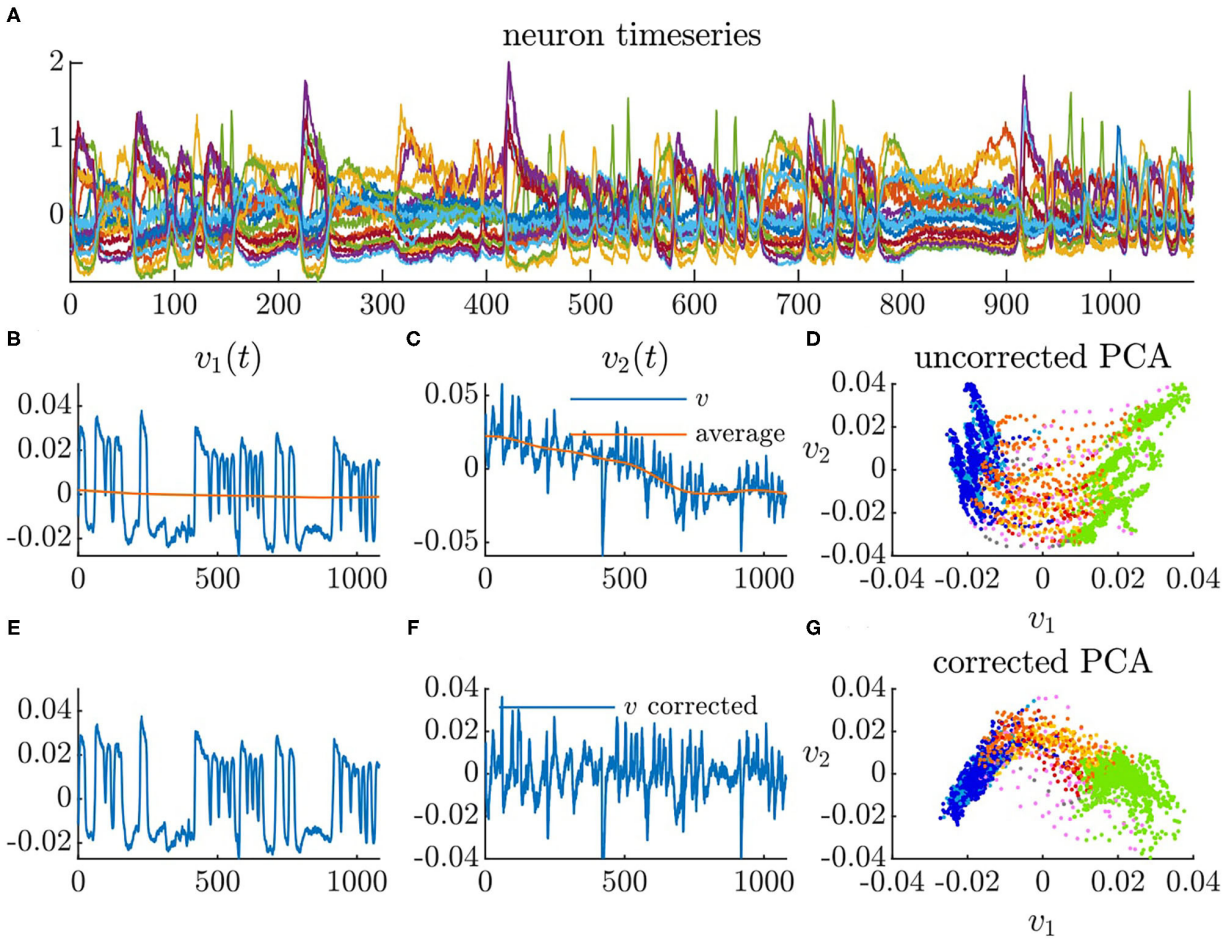
Principal component analysis of neural activity. **(A)** Calcium imaging timeseries mean centered (time in seconds). **(B)** Timeseries of first principal component with moving average. **(C)** Timeseries of second principal component with moving average. **(D)** Neural activity in PCA space using uncorrected PCA. **(E)** Timeseries of first principal component with moving average subtracted. **(F)** Timeseries of second principal component with moving average subtracted. **(G)** Neural activity in PCA space using corrected PCA.

Each of the five *C. elegans* datasets contains calcium imaging from 107 to 131 neurons. We performed PCA on the timeseries of each *C. elegans* neural activity, which is calculated as the time derivatives of the normalized Ca^2+^ traces (Δ*F*/*F*) (Kato et al., 2015). We used the first two PCA modes to construct a dynamical systems model. The first two PCA modes capture 18–23% of the total variance in each dataset meaning that our model only represents the dominant neural activity and is excluding secondary activity. Bleaching causes the calcium imaging signals to dampen over time in each timeseries (Kato et al., 2015). Because we are only interested in transitions between behaviors that are known to be faster timescale (Kaplan et al., 2020), we correct for this by subtracting a long-timescale moving average from each principal component. Figure 5A shows the mean centered neural activity of a single *C. elegans*. Figures 5B–D shows the first two principal components of the neural activity without drift correction while Figures 5E–G shows the first two principal components with drift correction.

### 4.2. Nonlinear Dynamical Systems Model

Nonlinear dynamical systems are ubiquitous in the engineering, physical and biological sciences for describing many complex phenomena observed in a diverse number of settings. Often, simple qualitative models with polynomial non-linearities are capable of providing remarkable insight into dynamical behaviors. The nonlinear pendulum, for instance, can be approximated by a Taylor series expansion to characterize the effects of frequency shifts and harmonic generation that is observed in practice. Inspired by well-studied non-linearities, we consider dynamical systems of the general form

(6) $$\begin{array}{r} \overset{∙}{\text{x}}=f\left(\text{x},\beta ,\gamma \right)+\text{B}u\left(t\right). \end{array}$$

We restrict our focus to polynomial equations with fixed points that can be determined analytically:

(7) $$\begin{array}{r} \;x^{\prime }=y\\ \;y^{\prime }=f\left(x\right)+\gamma y+u\left(t\right)\\ f\left(x\right)=a\overset{n}{\underset{i=1}{\prod }}\left(x-r_{i}\right) \end{array}$$

where *f*(*x*) is a polynomial with a leading coefficient *a* and roots *r*_*i*_. γ is the damping parameter. This is a second order nonlinear differential equation which can be expressed as *x*″−*f*(*x*)−γ*x*′ = 0. If γ = 0, the system is undamped and the differential equation becomes *x*″ = *f*(*x*) which has an analytical solution. Often however, the solutions are exceedingly complex and it is preferable to take a qualitative approach. We choose a system of this form as the fixed points can be easily placed and assigned a stability type (e.g., saddles, sources, sinks, or centers) through parameter selection. All fixed points lie on the x-axis and are placed and manipulated by varying our polynomial roots *r*_*i*_, while fixed point stability types are assigned by manipulating γ and *a* for a given set of roots *r*_*i*_. Two stable fixed points have been identified in the *C. elegans* dynamics, suggesting a cubic dynamical system. Additional features of the data and how they can be translated into a nonlinear dynamical system are described in Table 3.

**Table 3:** Features exhibited by *C. elegans* neural activity paired with corresponding dynamical system features.

***C. elegans***	**Dynamical System**
Two stable fixed points	Globally stable system with two sinks
System functions with variability	System behavior remains qualitatively constant under small parameter perturbations
Trajectories contain stochasticity	System behavior remains qualitatively constant with the addition of noise
Fixed point locations drift	Behavior remains qualitatively constant despite deformations and shifts to the system
Trajectories tend to follow set paths	System path variability set with damping term

### 4.3. Model Fitting

We fit the parameters (*P* = [β* γ σ u*_3,4_ *u*_5,6_ *dt*]) of our general model (Equation 4) to each *C. elegans* low-dimensional activity by minimizing the error ∫|*E*(*t*)|*dt* where *E*(*t*) = *v*_1_(*t*)−*x*(*t*). Parameter β is the location of the saddle fixed point and controls the relative stability of forward and reversal states, γ is the damping parameter, and σ is the level of stochasticity in the system. Control signal *u*(*t*) = *u*_3,4_ during states 3 and 4 (dorsal and ventral turns) and *u*(*t*) = *u*_5,6_ during states 5 and 6 (rev 1 and rev 2 transitions). The behavioral state timeseries has been determined by Kato et al. (2015). Parameter *dt* scales the model timesteps so that they fit the measurement intervals of the calcium imaging data. This is a non-convex optimization problem. We first perform a random search over the parameter space until the model performs most transitions. We then continue optimizing via MATLAB's fminsearch function. The random search resulted in parameters *P* = [0.1−1.5 0.06 0.5−0.7 0.3] which we used as the initial condition for the fminsearch function. We optimized for >200 interactions for each model fit. While this method finds a suitable collection of parameters that execute the transitions observed in the data (Figure 3), it does not guarantee the optimal solution will be found.

### 4.4. Robustness of Results to Parameter Variations

**Figure 6:**
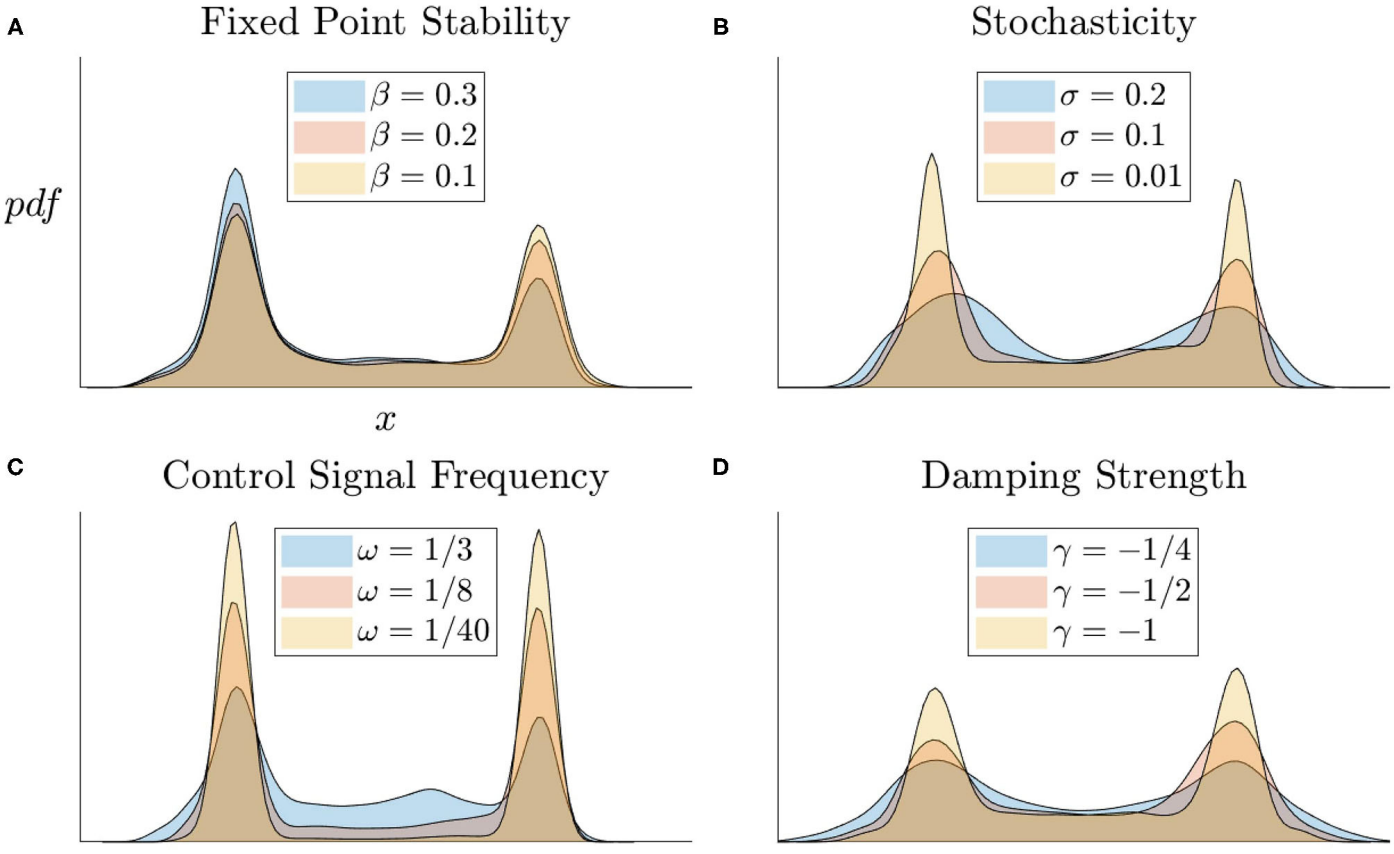
State distributions of nonlinear models for various parameter regimes. **(A)** Fixed point relative locations affects their stability. **(B)** Increasing levels of Brownian motion (σ) increases the variation about the fixed points. **(C)** More frequent control signals more evenly distributes the time spent in stable vs. transitional states. **(D)** Stronger damping in the system keeps trajectories close to fixed points.

We observe how modifying other system parameters affect the state distribution of the nonlinear system's activity under randomly generated control signals. In Figure 6A, we vary the right fixed point's region of stability by moving the location of the middle fixed point β. We observe the system spends less time at the right fixed point with a smaller stability region. In Figure 6B, we increase the level of Brownian motion (σ) in the system and observe the variability increases in the distributions as a result. In Figure 6C, we observe that increasing the control signal frequency increases the amount of time spend in a transitional state. Figure 6D shows that increasing the damping strength decreases the distribution variability. Observing these parameter variations holistically, we see that the nonlinear model is able to perform the task of switching between fixed points under a wide range of parameter values which insures the integrity of the system and indicates that *C. elegans* dynamics, if comparable to this model, should be able to operate robustly and stably under a diverse array of environments and internal states.

## Data Availability Statement

Publicly available datasets were analyzed in this study. This data can be found here: https://osf.io/a64uz/ Repositories: Kato2015, Skora2018.

## Author Contributions

MM was the primary producer of the model with input from CF and JK. The figures for the manuscript were created by MM and the content written by MM, CF, and JK. All authors contributed to the article and approved the submitted version.

## Conflict of Interest

The authors declare that the research was conducted in the absence of any commercial or financial relationships that could be construed as a potential conflict of interest.
